# Supplementary material for: Hair Follicle Targeting and Dermal Drug Delivery with Curcumin Drug Nanocrystals—Essential Influence of Excipients
Source: Nanomaterials (Basel). 2020 Nov 23;10(11):2323. doi: 10.3390/nano10112323 (PMC7700376; doi:10.3390/nano10112323)
Supplement: Supplementary file 1 [file nanomaterials-10-02323-s001.pdf]

Supplementary Materials: Hair Follicle Targeting and Dermal Drug Delivery with Curcumin Drug Nanocrystals—Essential Influence of Excipients

Olga Pelikh and Cornelia M. Keck *

S1: Untreated Skin.

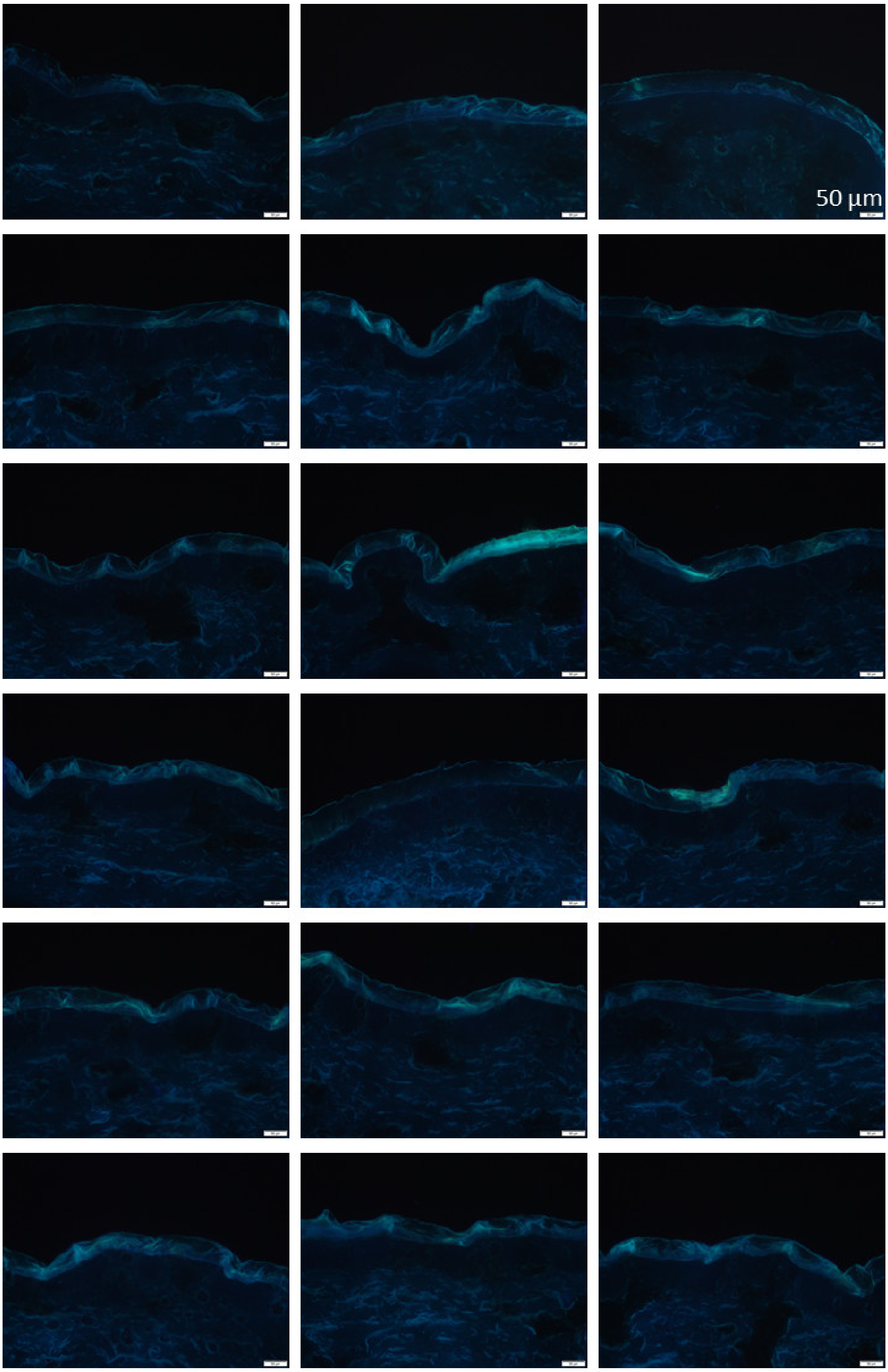

Figure S1. Skin sections of untreated skin. Magnification: 200 fold.

S2: Skin Treated with Nanosuspension without Additives.

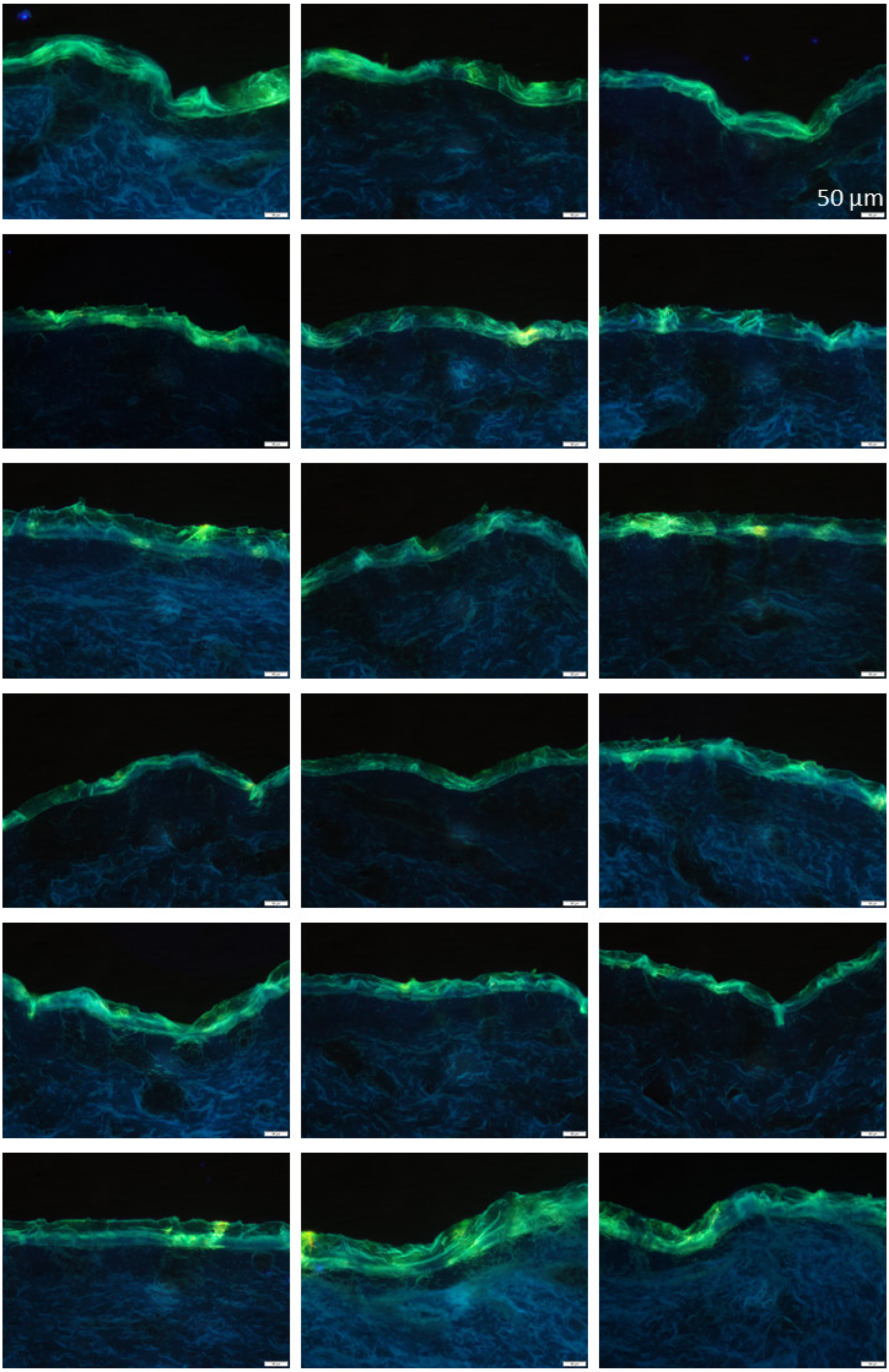

Figure S2. Skin sections of skin treated with nanosuspension without additives. Magnification: 200 fold.

S3: Skin Treated with Nanosuspension with 2% Glycerol.

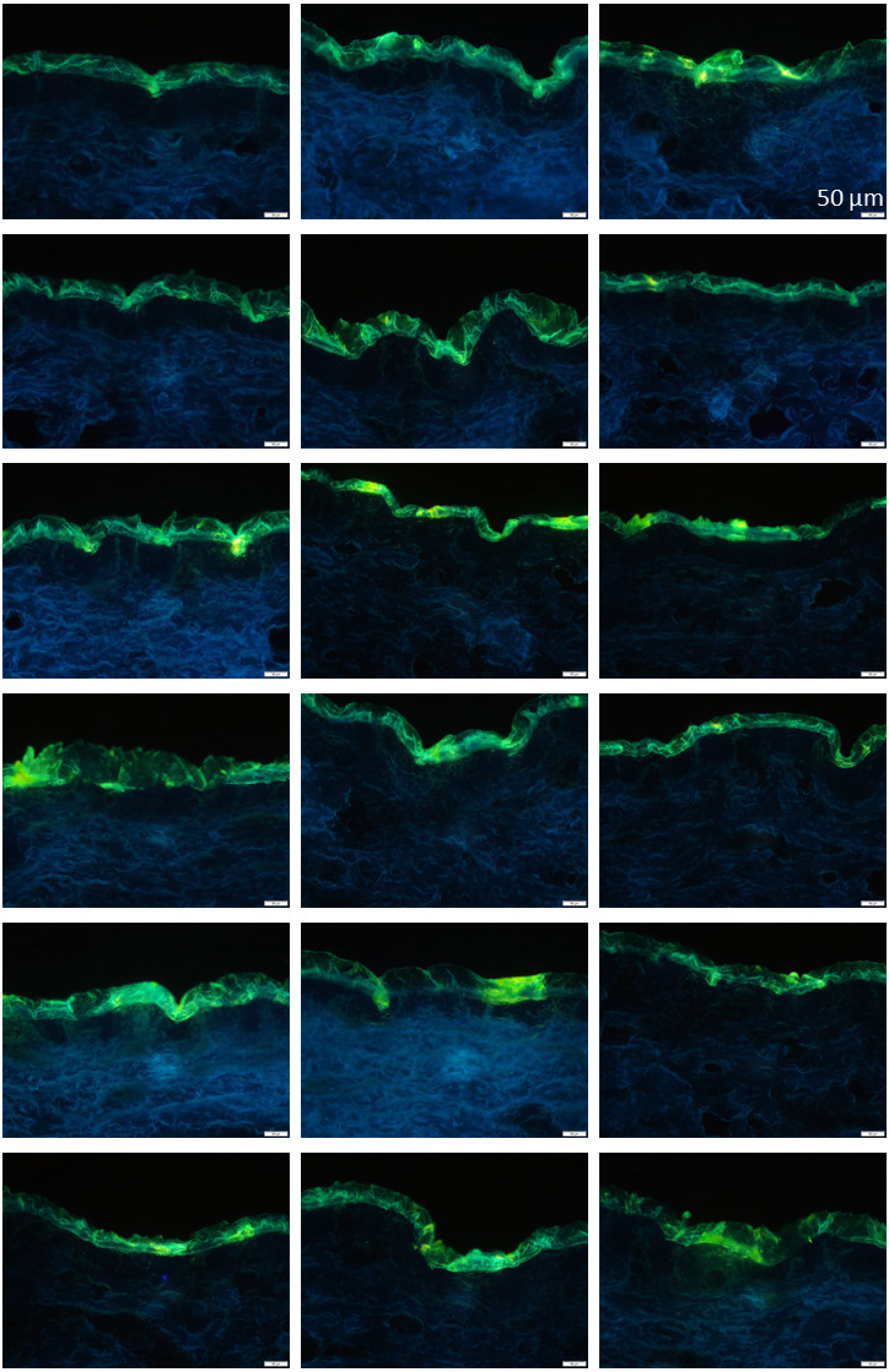

Figure S3. Skin sections of skin treated with nanosuspension contained 2% glycerol. Magnification: 200 fold.

S4: Skin Treated with Nanosuspension with 5% Glycerol.

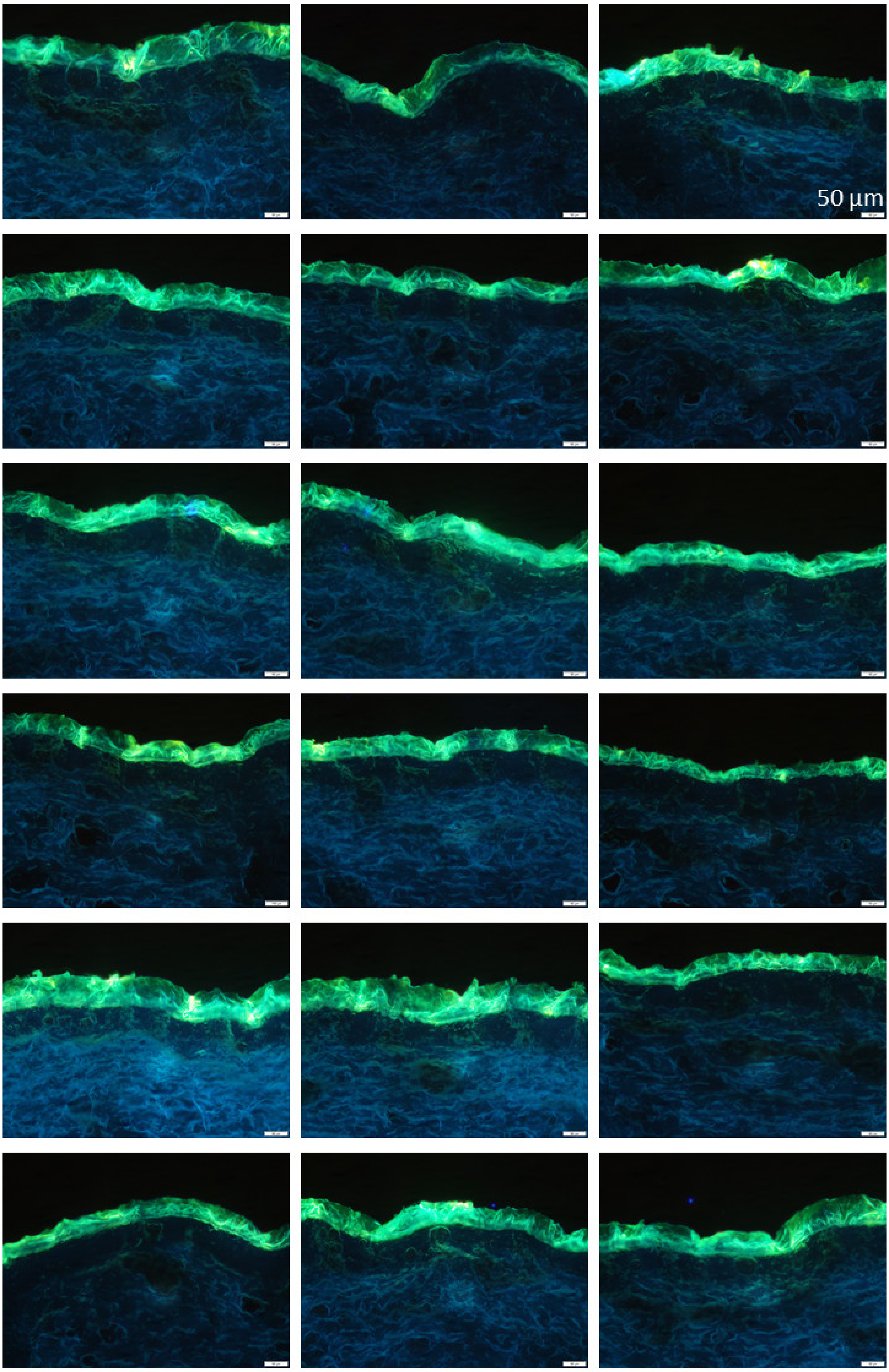

Figure S4. Skin sections of skin treated with nanosuspension contained 5% glycerol. Magnification: 200 fold.

S5: Skin Treated with Nanosuspension with 5% Urea.

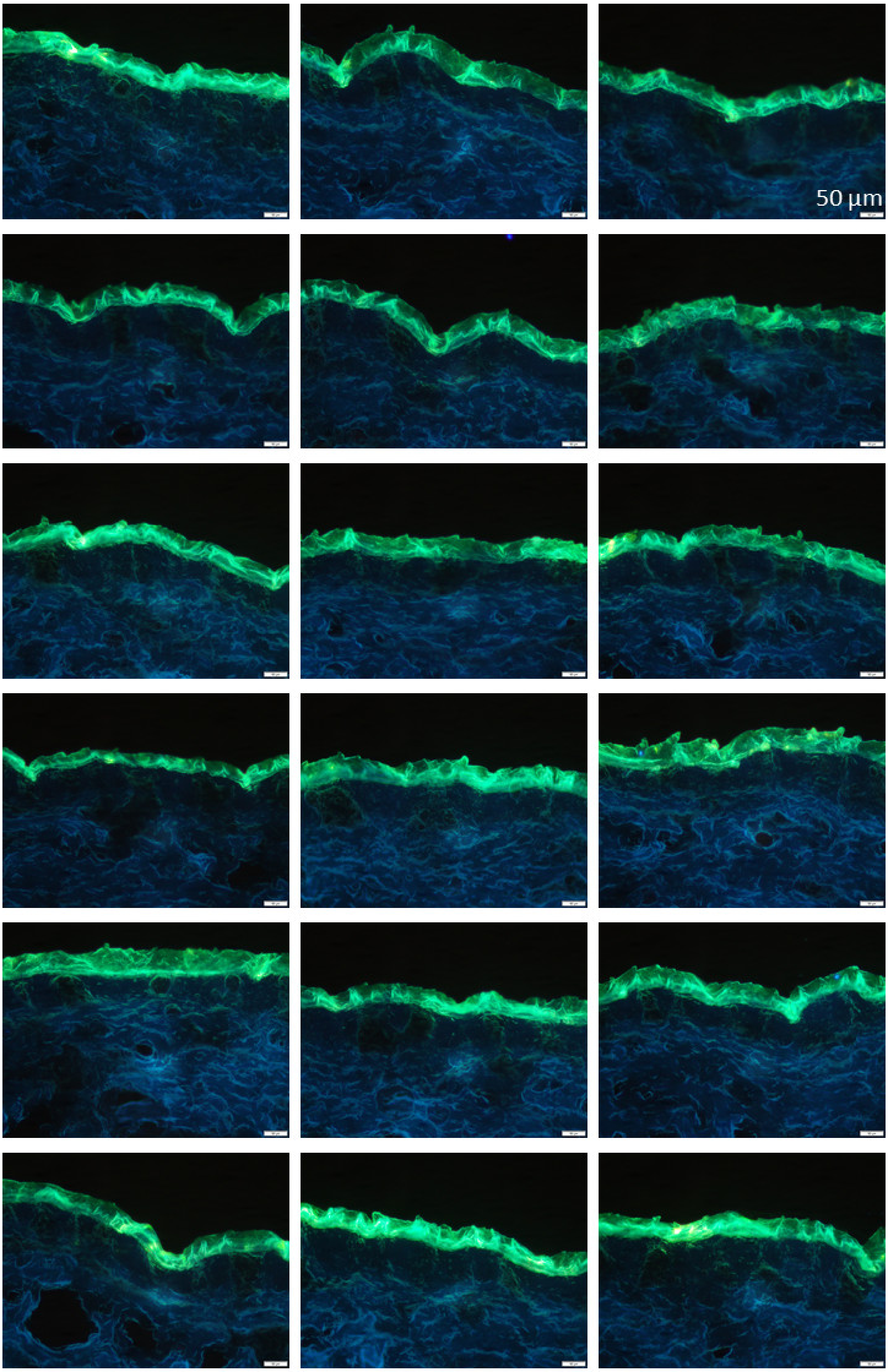

Figure S5. Skin sections of skin treated with nanosuspension contained 5% urea. Magnification: 200 fold.

S6: Skin Treated with Nanosuspension with 10% Urea.

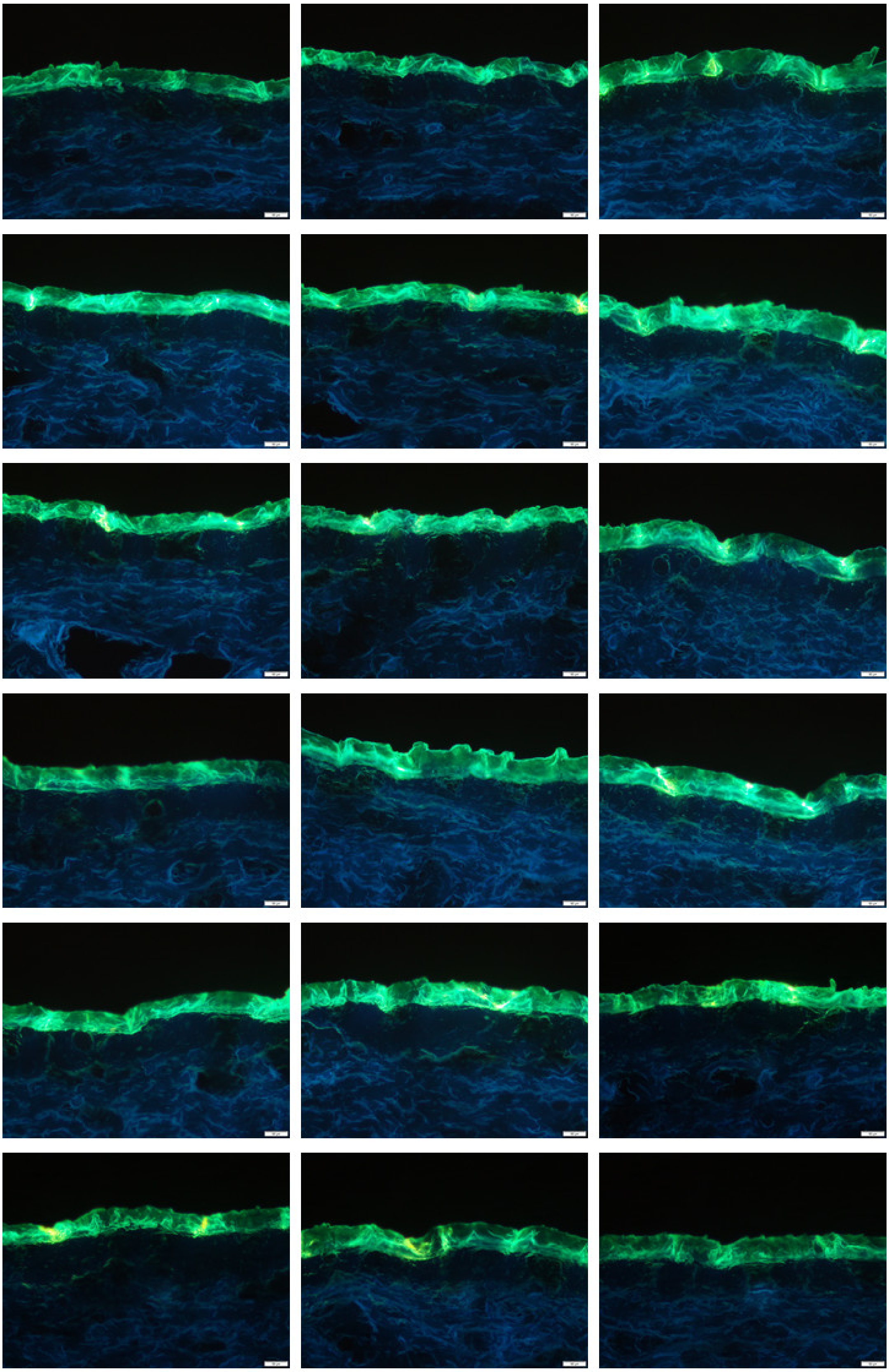

Figure S6. Skin sections of skin treated with nanosuspension contained 10% urea. Magnification: 200 fold.

S7: Skin Treated with Nanosuspension with 5% Propylene Glycol.

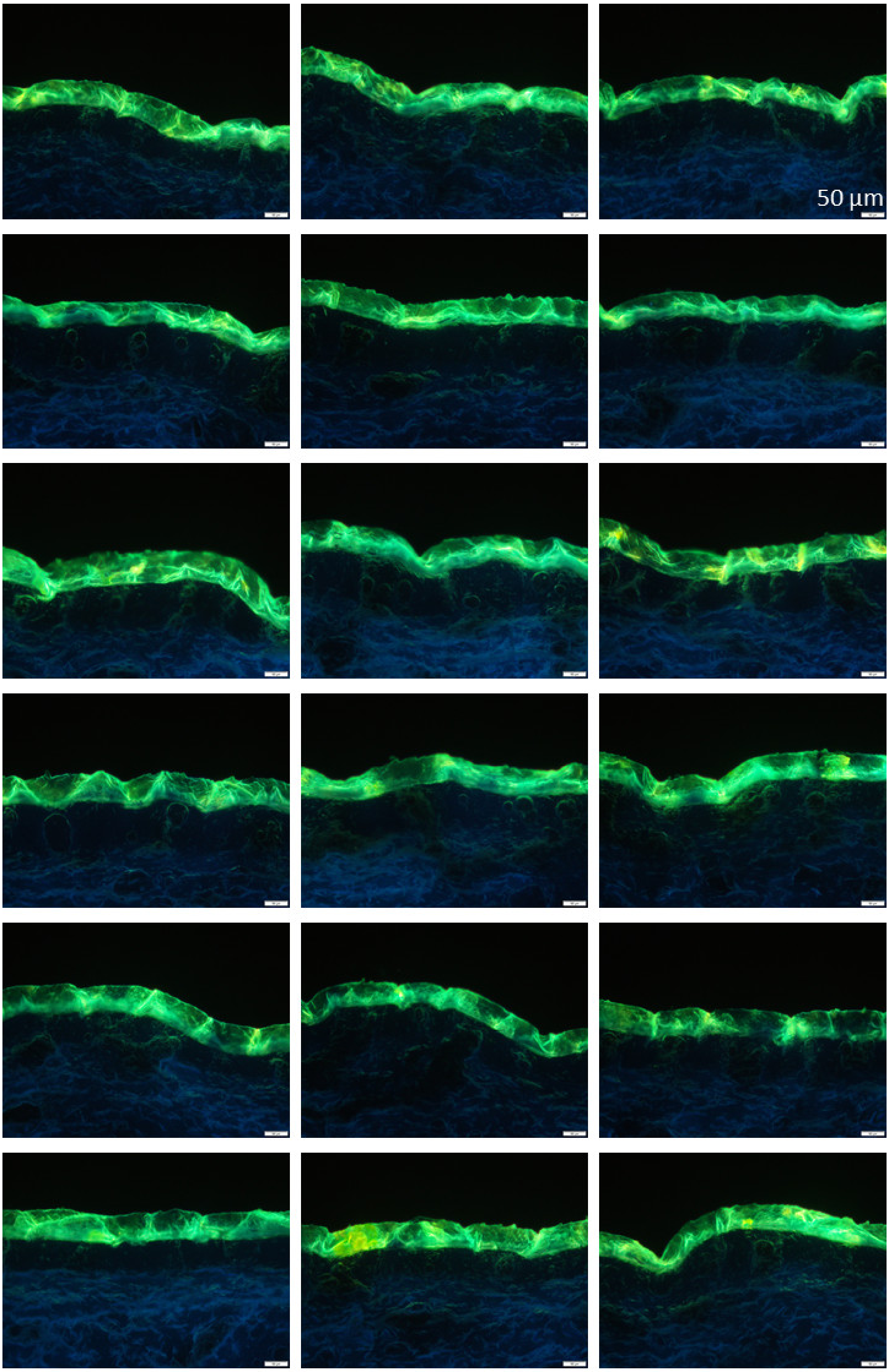

Figure S7. Skin sections of skin treated with nanosuspension contained 5% propylene glycol. Magnification: 200 fold.

S8: Skin Treated with Nanosuspension with 2% Ethanol.

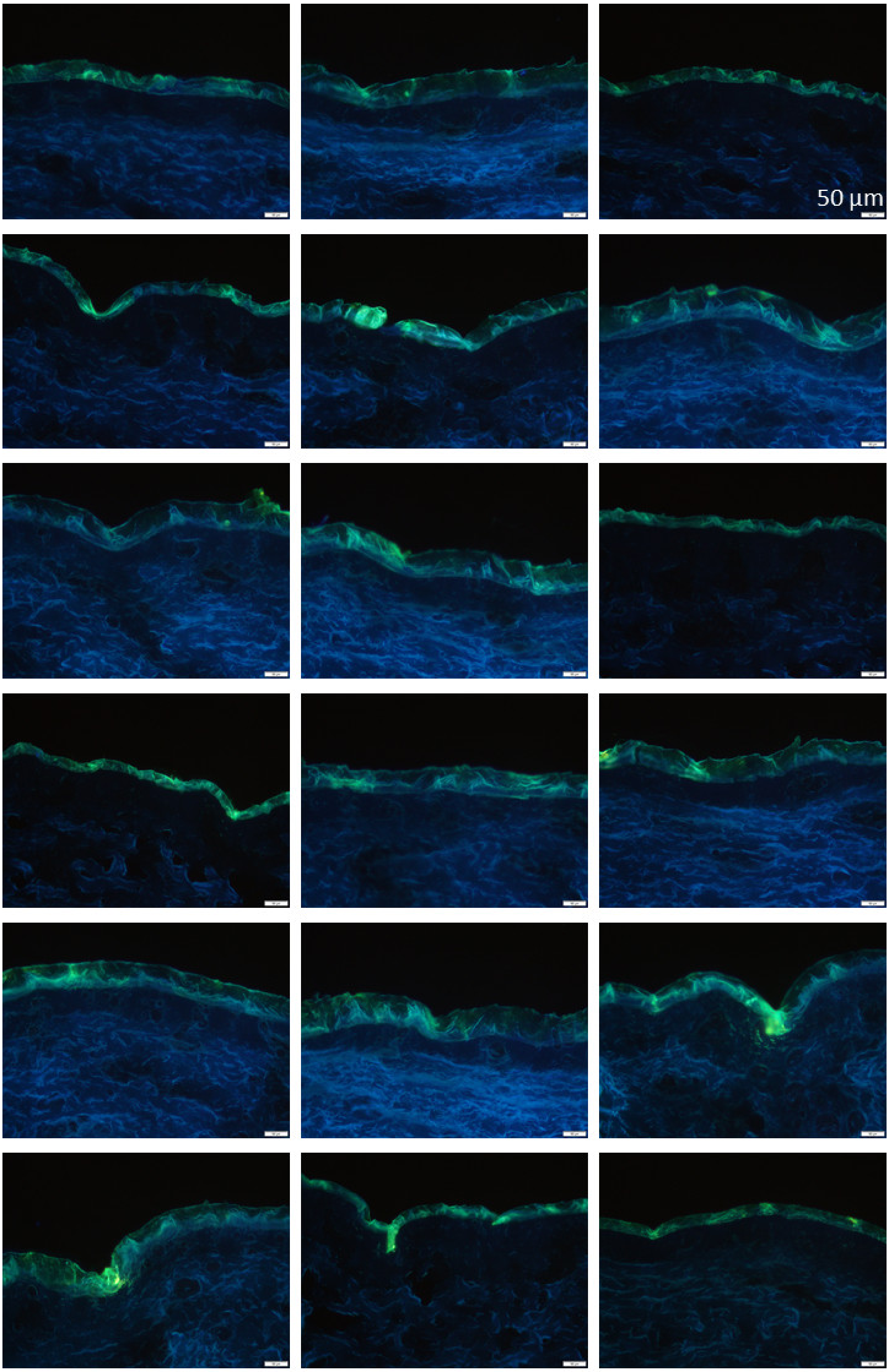

Figure S8. Skin sections of skin treated with nanosuspension contained 2% ethanol. Magnification: 200 fold.

S9: Skin Treated with Nanosuspension with 2% Olive Oil.

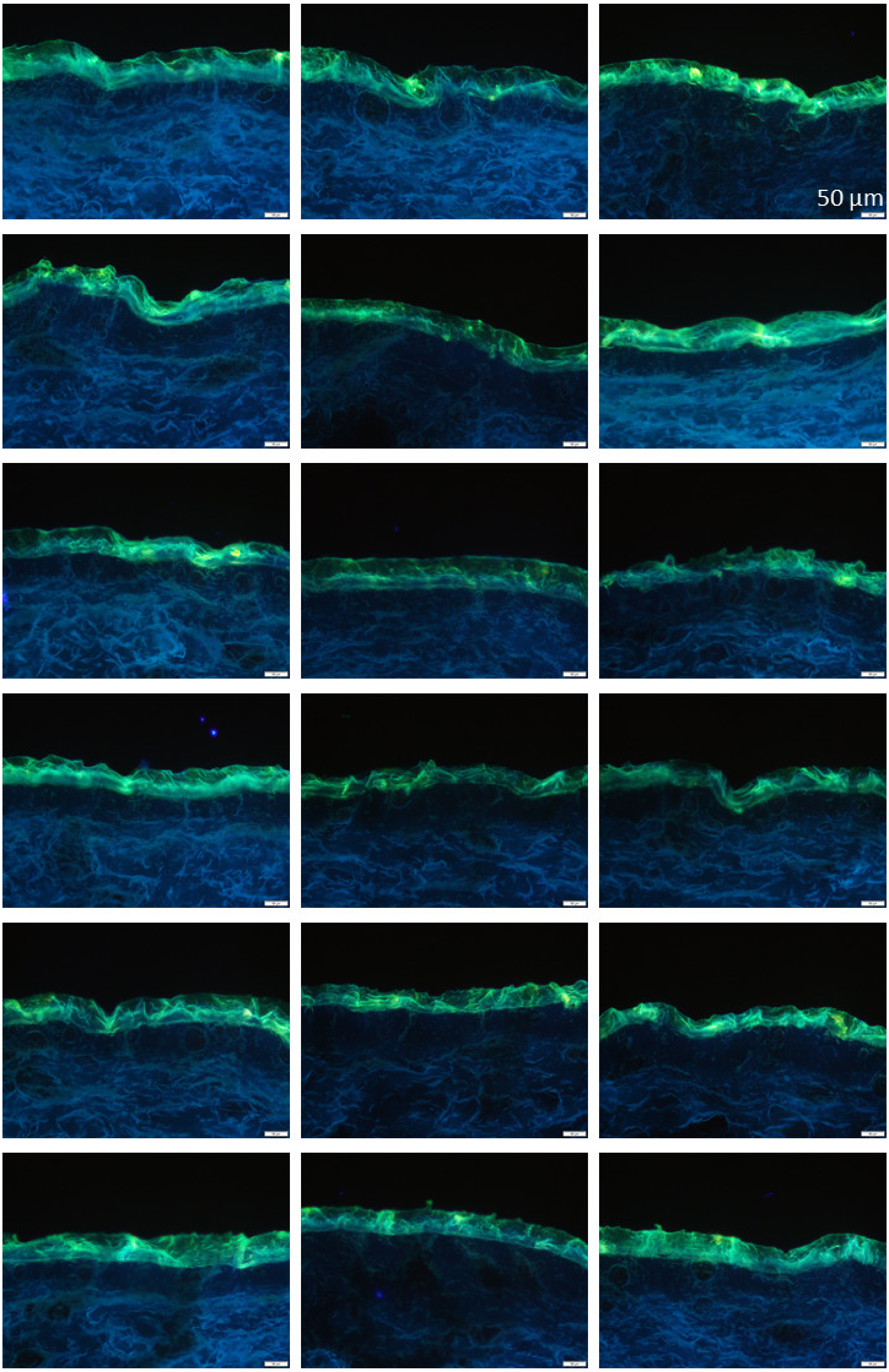

Figure S9. Skin sections of skin treated with nanosuspension contained 2% olive oil. Magnification: 200 fold.

S10: Hair Follicles Treated with Curcumin Nanocrystals without Additives.

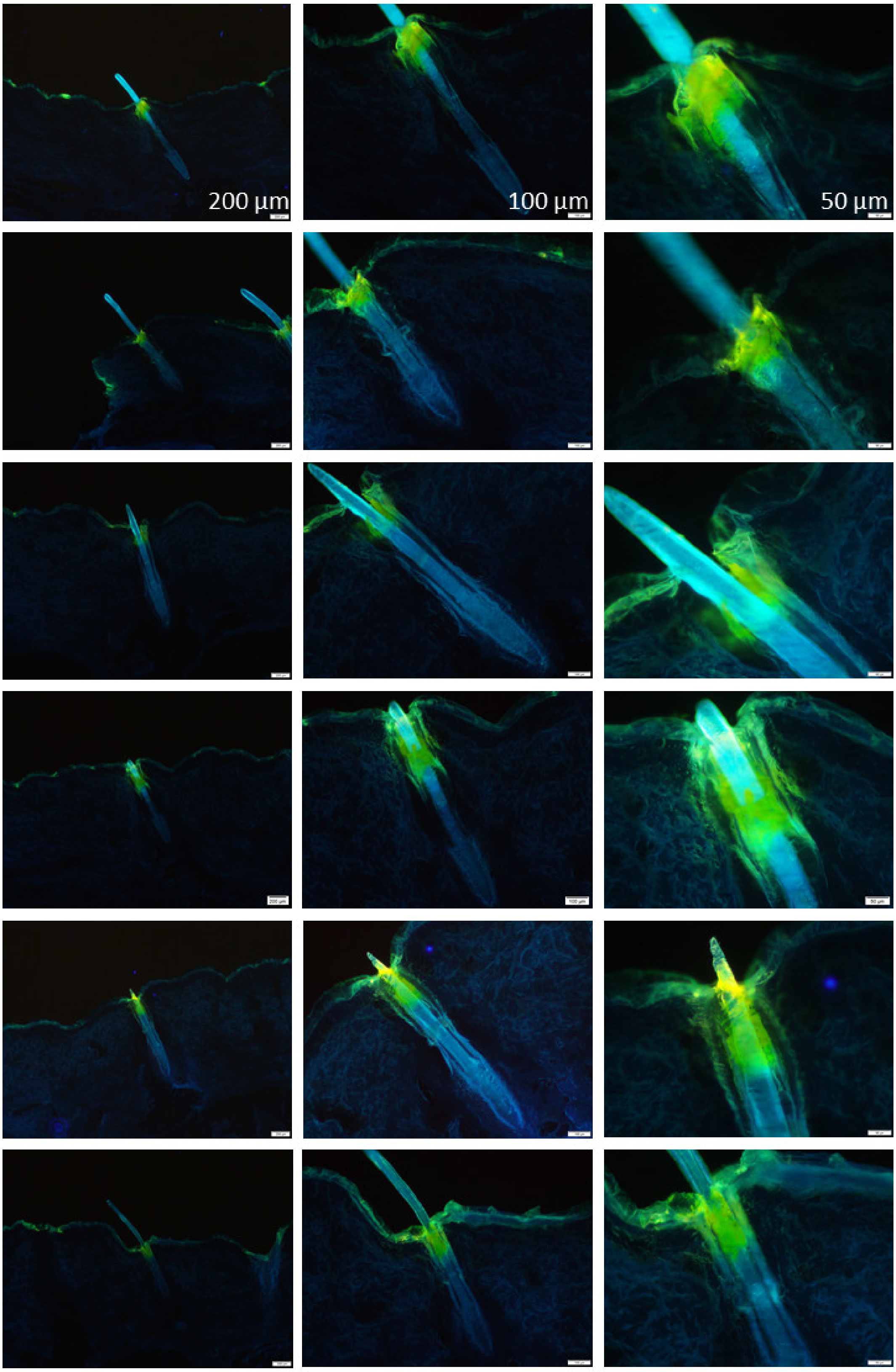

Figure S10. Hair follicles treated with nanosuspension without additives. Left column: images taken at 40fold magnification. Middle column: images taken at 100 fold magnification. Right column: images taken at 200 fold magnification.

S11: Hair Follicles Treated with Curcumin Nanocrystals with 2% Glycerol.

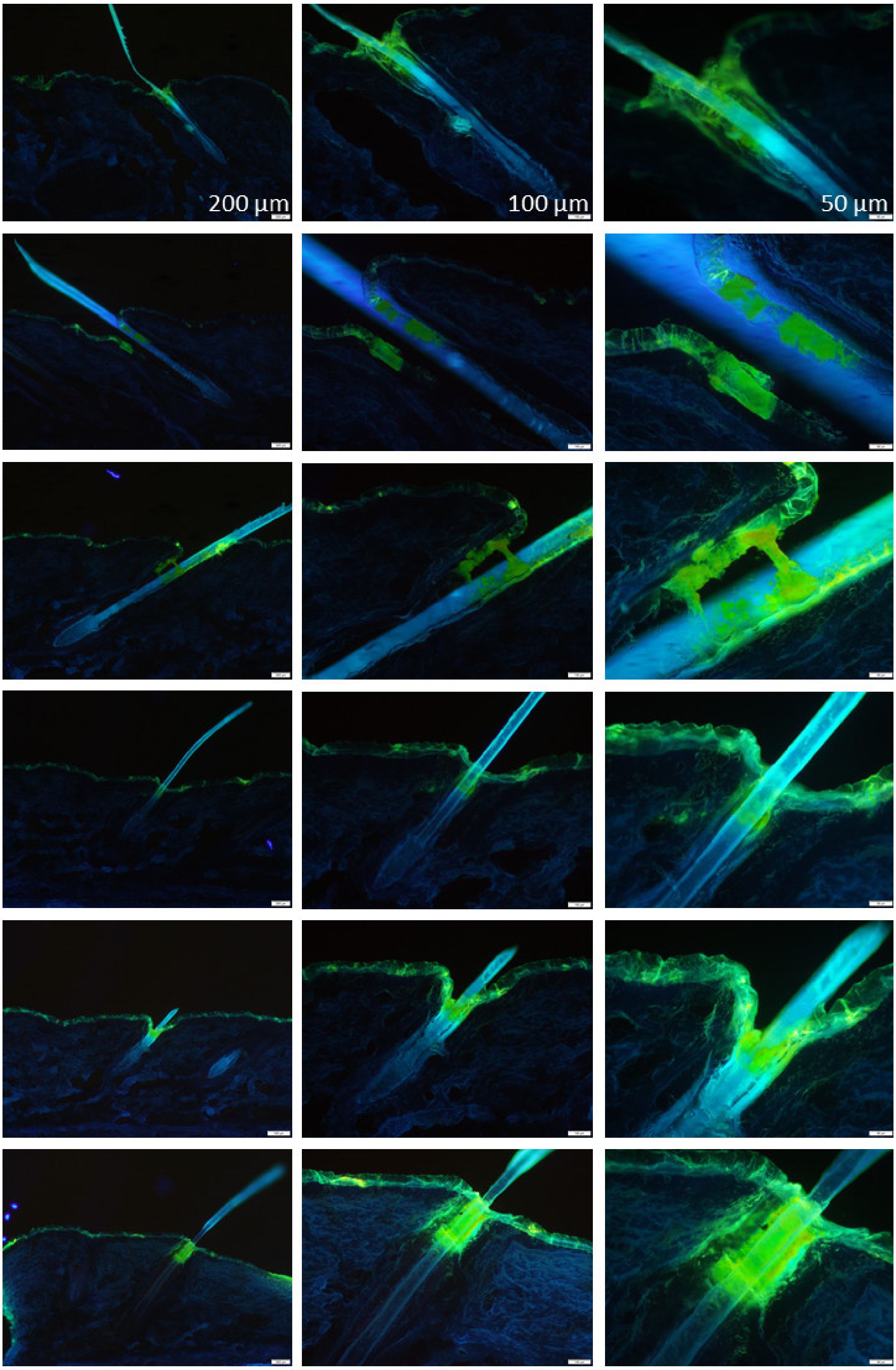

Figure S11. Hair follicles treated with nanosuspension with 2% glycerol. Left column: images taken at 40 fold magnification. Middle column: images taken at 100 fold magnification. Right column: images taken at 200 fold magnification.

S12: Hair Follicles Treated with Curcumin Nanocrystals with 5% Glycerol.

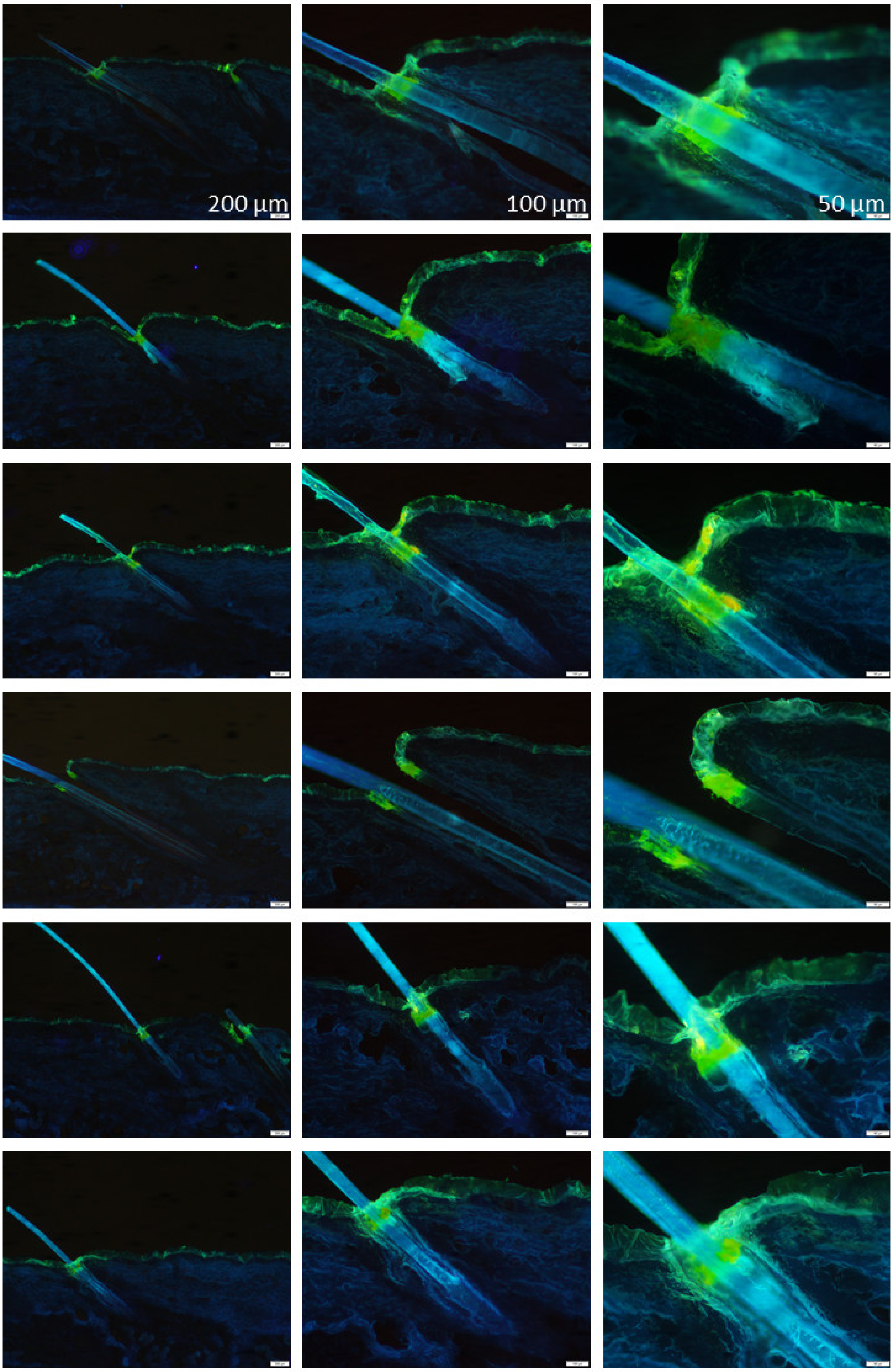

Figure S12. Hair follicles treated with nanosuspension with 5% glycerol. Left column: images taken at 40 fold magnification. Middle column: images taken at 100fold magnification. Right column: images taken at 200 fold magnification.

S13: Hair Follicles Treated with Curcumin Nanocrystals with 5% Urea.

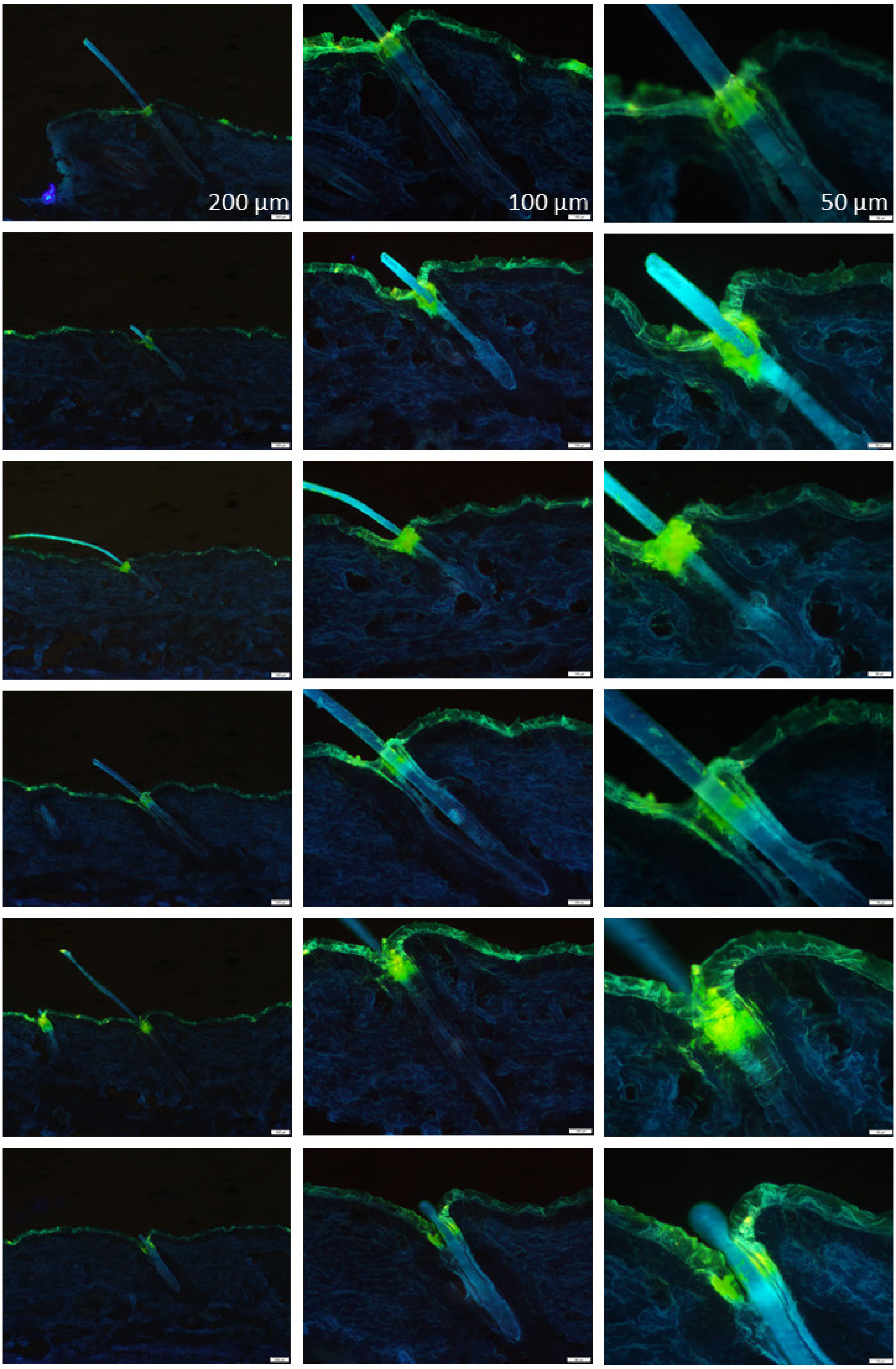

Figure S13. Hair follicles treated with nanosuspension with 5% urea. Left column: images taken at 40fold magnification. Middle column: images taken at 100 fold magnification. Right column: images taken at 200 fold magnification.

S14: Hair Follicles Treated with Curcumin Nanocrystals with 10% Urea.

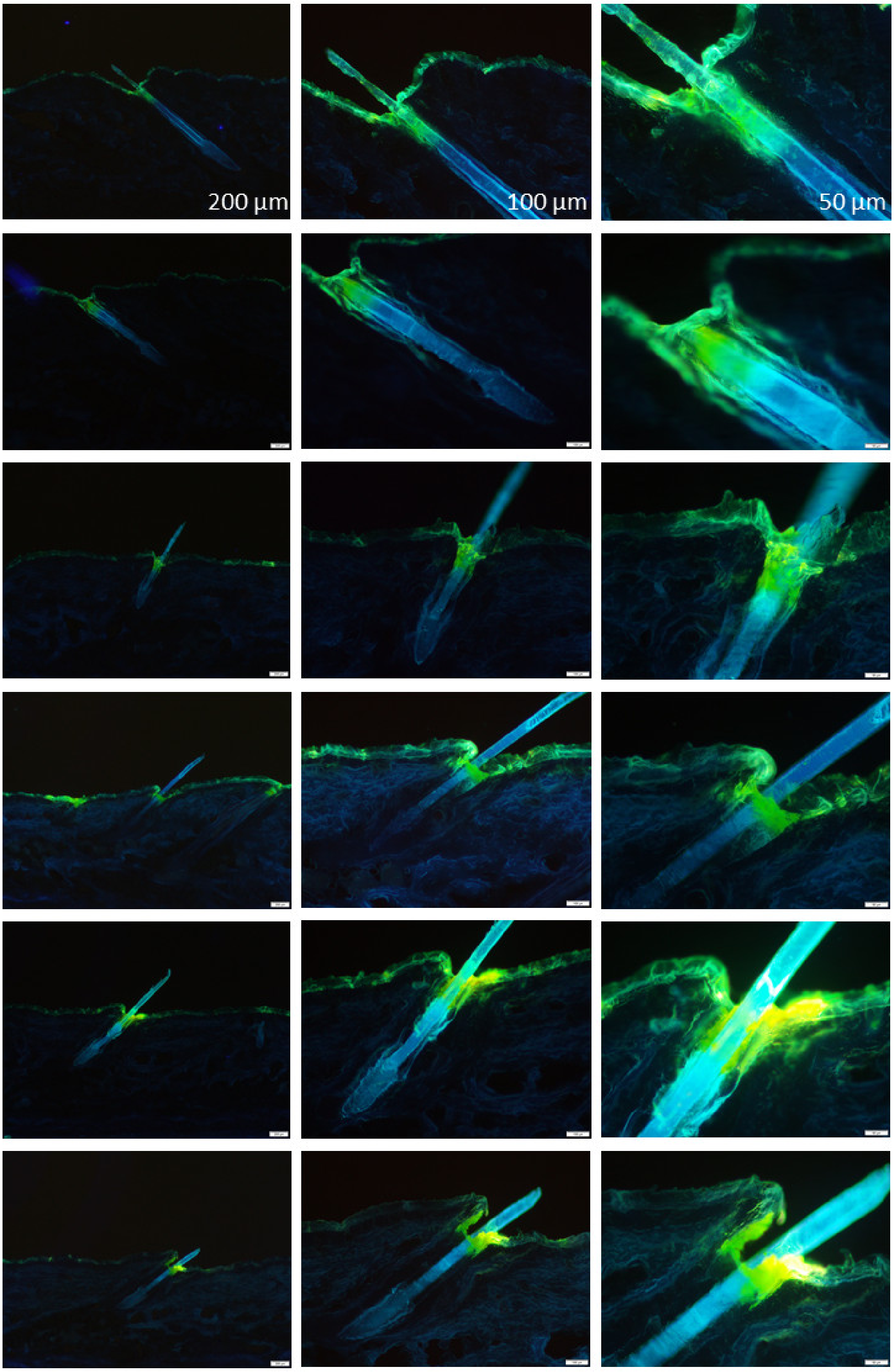

Figure S14. Hair follicles treated with nanosuspension with 10% urea. Left column: images taken at 40 fold magnification. Middle column: images taken at 100 fold magnification. Right column: images taken at 200 fold magnification.

S15: Hair Follicles Treated with Curcumin Nanocrystals without Additives after 14 days Storage.

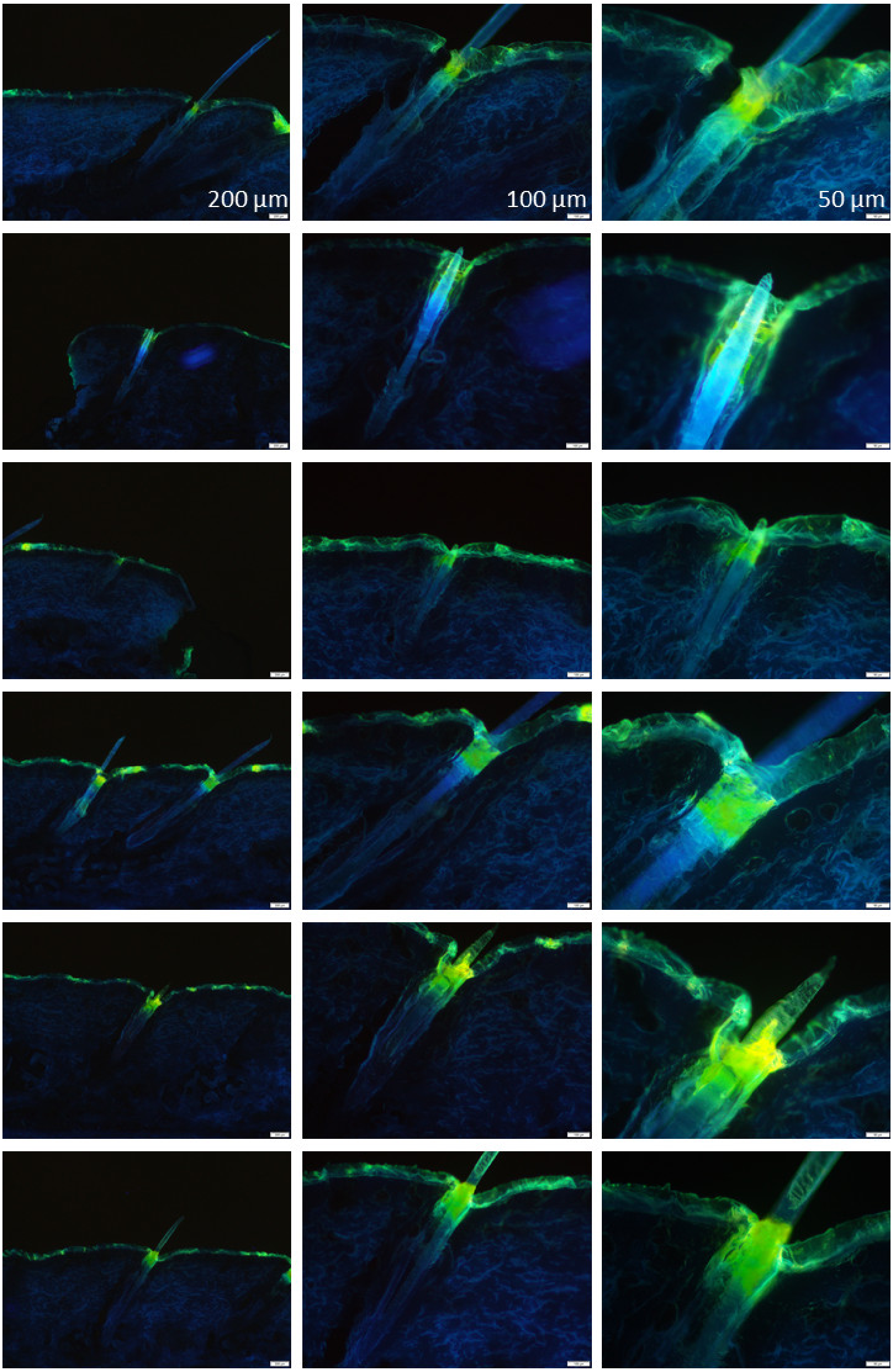

Figure S15. Hair follicles treated with nanosuspension without additives after 14 days storage. Left column: images taken at 40fold magnification. Middle column: images taken at 100 fold magnification. Right column: images taken at 200 fold magnification.

S16: Hair Follicles Treated with Curcumin Nanocrystals with 5% Propylene Glycol.

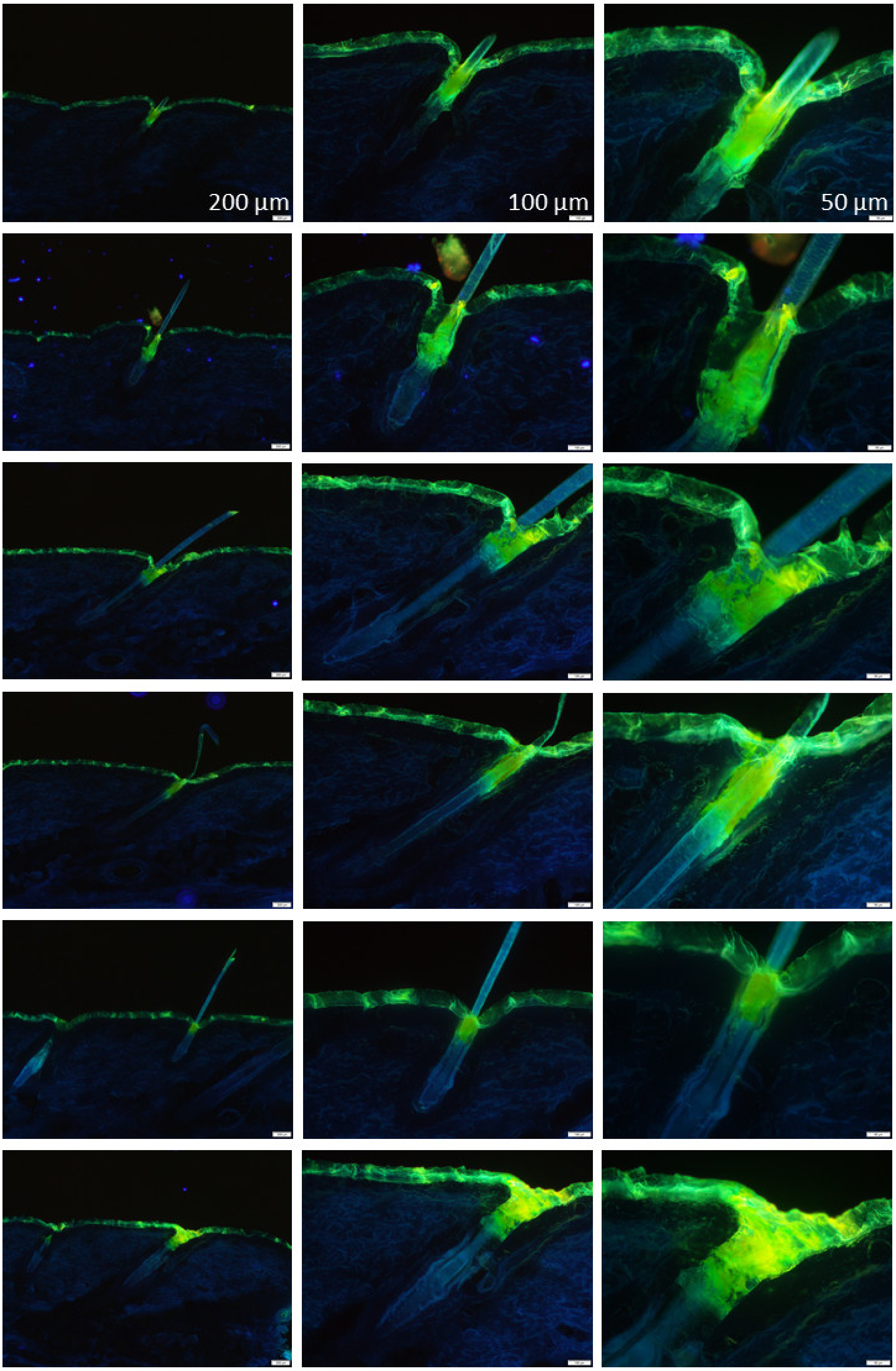

Figure S16. Hair follicles treated with nanosuspension with 5% propylene glycol. Left column: images taken at 40 fold magnification. Middle column: images taken at 100 fold magnification. Right column: images taken at 200 fold magnification.

S17: Hair Follicles Treated with Curcumin Nanocrystals with 2% Ethanol.

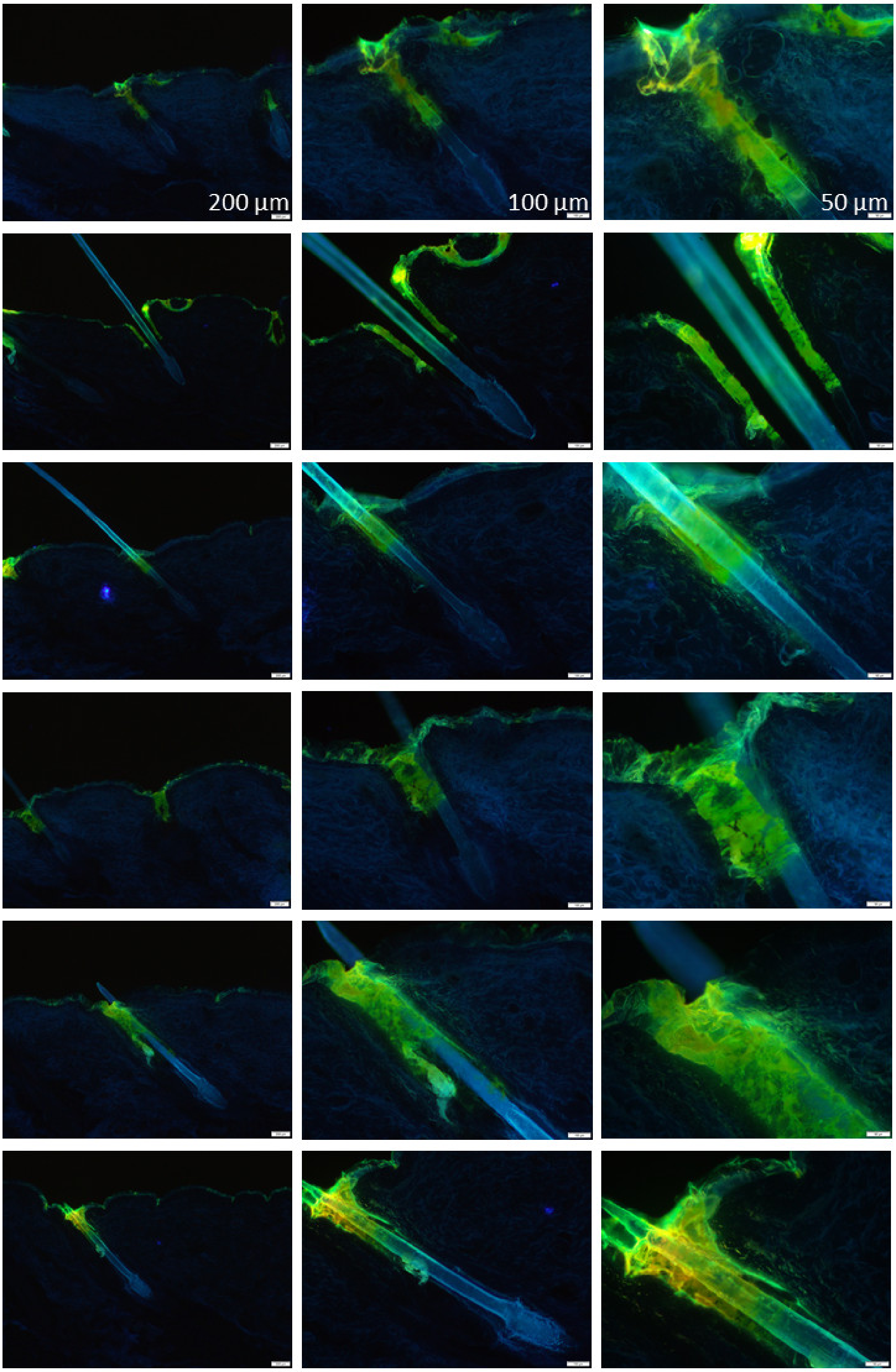

Figure S17. Hair follicles treated with nanosuspension with 2% ethanol. Left column: images taken at 40fold magnification. Middle column: images taken at 100 fold magnification. Right column: images taken at 200 fold magnification.

S18: Hair Follicles Treated with Curcumin Nanocrystals with 2% Olive Oil.

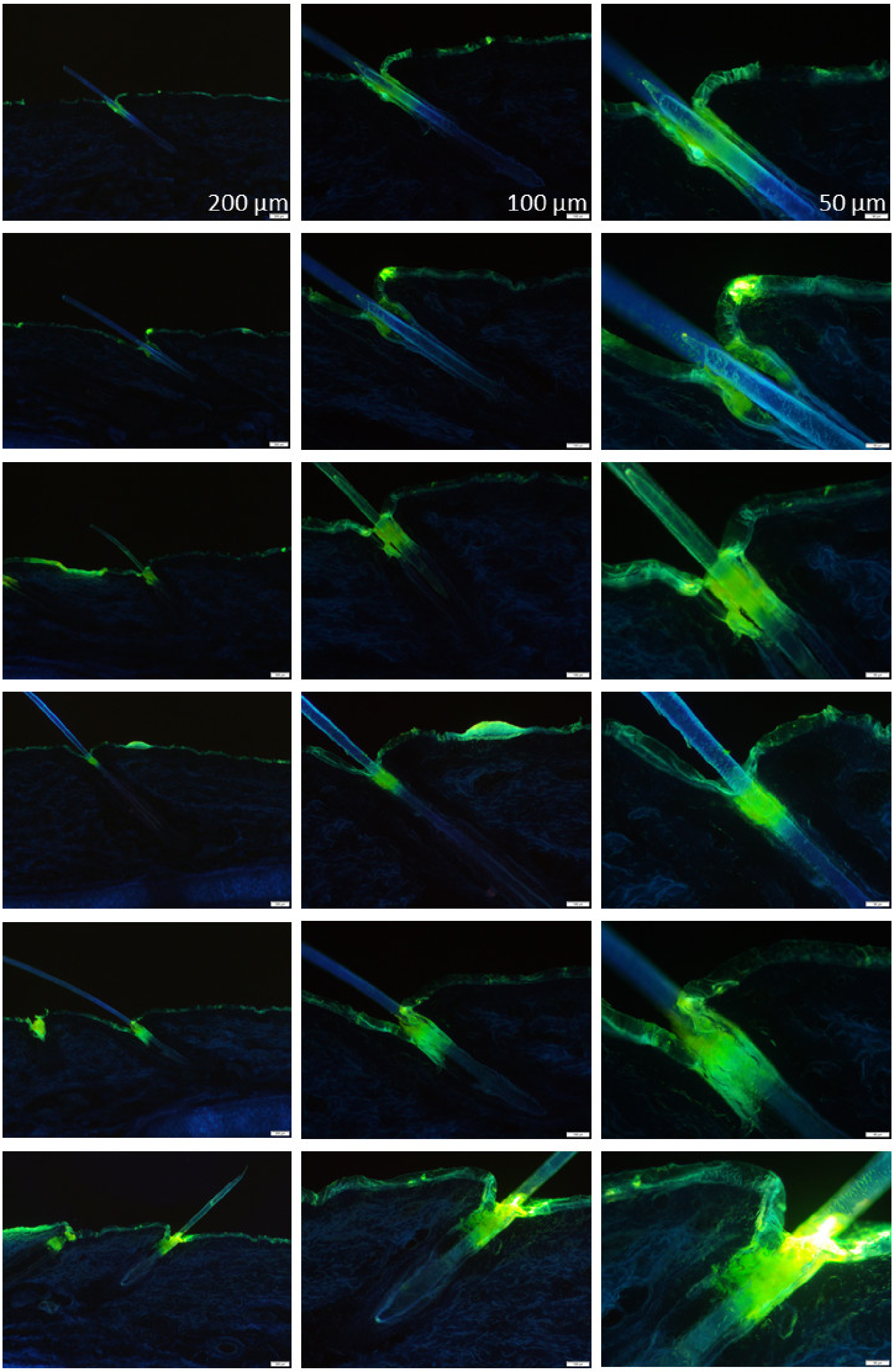

Figure S18. Hair follicles treated with nanosuspension with 2% olive oil. Left column: images taken at 40 fold magnification. Middle column: images taken at 100 fold magnification. Right column: images taken at 200 fold magnification.

© 2020 by the author. Licensee MDPI, Basel, Switzerland. This article is an open access article distributed under the terms and conditions of the Creative Commons Attribution (CC BY) license (http://creativecommons.org/licenses/by/4.0/).
